# A unique arginine cluster in PolDIP2 enhances nucleotide binding and DNA synthesis by PrimPol

**DOI:** 10.1093/nar/gkab049

**Published:** 2021-02-03

**Authors:** Kazutoshi Kasho, Gorazd Stojkovič, Cristina Velázquez-Ruiz, Maria Isabel Martínez-Jiménez, Mara Doimo, Timothée Laurent, Andreas Berner, Aldo E Pérez-Rivera, Louise Jenninger, Luis Blanco, Sjoerd Wanrooij

**Affiliations:** Department of Medical Biochemistry and Biophysics, Umeå University, 90187 Umeå, Sweden; Department of Medical Biochemistry and Biophysics, Umeå University, 90187 Umeå, Sweden; Centro de Biologia Molecular Severo Ochoa, E-28049 Madrid, Spain; Centro de Biologia Molecular Severo Ochoa, E-28049 Madrid, Spain; Department of Medical Biochemistry and Biophysics, Umeå University, 90187 Umeå, Sweden; Department of Medical Biochemistry and Biophysics, Umeå University, 90187 Umeå, Sweden; Department of Medical Biochemistry and Biophysics, Umeå University, 90187 Umeå, Sweden; Centro de Biologia Molecular Severo Ochoa, E-28049 Madrid, Spain; Department of Medical Biochemistry and Cell Biology, University of Gothenburg, 405 30 Gothenburg, Sweden; Centro de Biologia Molecular Severo Ochoa, E-28049 Madrid, Spain; Department of Medical Biochemistry and Biophysics, Umeå University, 90187 Umeå, Sweden

## Abstract

Replication forks often stall at damaged DNA. To overcome these obstructions and complete the DNA duplication in a timely fashion, replication can be restarted downstream of the DNA lesion. In mammalian cells, this repriming of replication can be achieved through the activities of primase and polymerase PrimPol. PrimPol is stimulated in DNA synthesis through interaction with PolDIP2, however the exact mechanism of this PolDIP2-dependent stimulation is still unclear. Here, we show that PrimPol uses a flexible loop to interact with the C-terminal ApaG-like domain of PolDIP2, and that this contact is essential for PrimPol's enhanced processivity. PolDIP2 increases primer-template and dNTP binding affinities of PrimPol, which concomitantly enhances its nucleotide incorporation efficiency. This stimulation is dependent on a unique arginine cluster in PolDIP2. Since the polymerase activity of PrimPol alone is very limited, this mechanism, where the affinity for dNTPs gets increased by PolDIP2 binding, might be critical for the *in vivo* function of PrimPol in tolerating DNA lesions at physiological nucleotide concentrations.

## INTRODUCTION

In all organisms, the bulk of genomic DNA replication is done by replicative DNA polymerases. These enzymes, characterized by their high processivity and fidelity, occasionally encounter obstacles on the DNA that significantly delay their progression, such as damaged DNA. If not alleviated, this can lead to fork collapse and genomic instability. In humans, genomic instability is tightly associated with disease, most notably cancer (1,2). In order to prevent genomic instability, cells have developed several DNA damage tolerance mechanisms that help the replication fork dealing with various disturbances and allow completion of the replication process.

**Figure 1.**
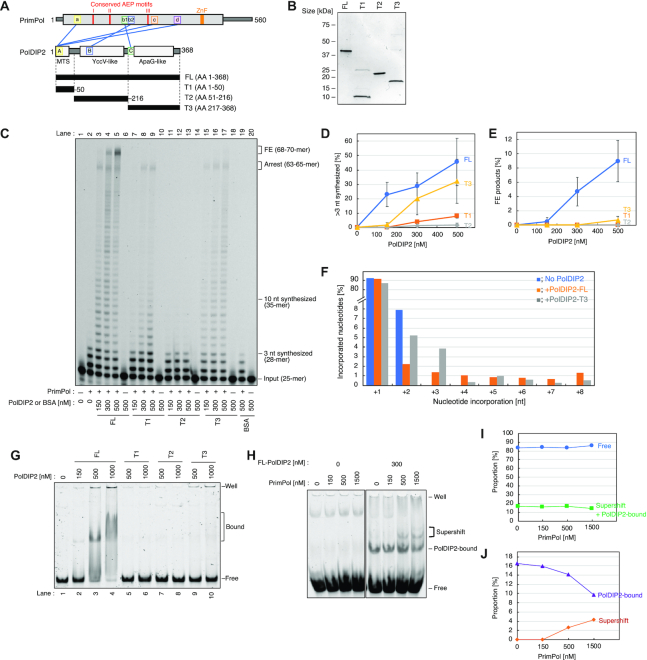
PrimPol DNA polymerase stimulation by the PolDIP2 ApaG-like domain. (**A**) Schematic presentation of the PrimPol–PolDIP2 interactions. The conserved AEP motifs of PrimPol (I, II and III) are indicated with red lines, and the zinc finger (ZnF) as an orange box. The PrimPol regions involved in PolDIP2 interaction are indicated as, ‘a’, ‘b1’, ‘b2’, ‘c’ and ‘d’, and the PolDIP2 regions involved in PrimPol interaction are indicated as, ‘A’, ‘B’ and ‘C’. Three PolDIP2 fragments, T1- (harboring the mitochondrial targeting signal (MTS)), T2- (YccV-like domainand) and T3-(ApaG-like domain) PolDIP2, were purified for this study. The interaction sites between PrimPol and PolDIP2, identified previously by crosslinking-mass spectrometry analysis (22), are shown with blue lines. (**B**) Purification of PolDIP2 fragments. Purified PolDIP2 fragment (300 ng) on SDS-PAGE gel stained with InstantBlue (Expedeon, UK). **(C-E)**Stimulation of PrimPol polymerase processivity by PolDIP2. (**C**) Reactions with 150 nM of PrimPol in the presence of 15 nM of 5′-TET-labeled primer/template DNA and the indicated amount of PolDIP2 fragments, or BSA (negative control). The positions of full extension (FE) products (68–70-mer), arrest site (63-65-mer), products extended for >10 nt (>35-mer) or for >3 nt (>28-mer), and input DNA (25-mer) are indicated. This experiment was repeated three times, and the percentage of (**D**) >3 nt synthesized and (**E**) full extension DNA products were quantified. The average values with standard deviations are shown with error bars. (**F**) Termination probabilities (y-axis) at positions between +1 and +8 under conditions where the product is derived from a single cycle DNA synthesis (single-hit conditions). The primer/template DNA (50 nM) was present at a 10-fold excess molarity over PrimPol (5 nM) to minimize the re-initiation of synthesis on the same template after an initial termination event. (**G**) PolDIP2-DNA binding assessed by EMSA. ‘Well’ – gel well. ‘Bound’ – DNA-PolDIP2 complexes. ‘Free’ – protein-free DNA. (**H-J**) FL-PolDIP2 can stimulate PrimPol binding to DNA. (**H**) ‘Well’ – gel well. ‘Supershift’ – the complexes dependent on both PrimPol and PolDIP2. ‘PolDIP2-bound’ – DNA–PolDIP2 complexes. ‘Free’ – protein-free DNA. Quantification of Figure 1H, showing (**I**) the proportion of protein-free or bound DNA and (**J**) DNA bound to PolDIP2 and PrimPol-dependent complexes.

One mode of DNA damage tolerance is translesion DNA synthesis (TLS), that uses specialized DNA polymerases, which at the expense of processivity and fidelity, can continue synthesis through the damaged sequence and therefore ensure the replication fork progression (3–5). Among various TLS polymerases, the primase/polymerase PrimPol has been shown to help both in nuclear and mitochondrial DNA replication fork progression (6-8). Additionally, PrimPol can also re-prime downstream of blocking lesions, thus re-initiating DNA replication (6,9–15). PrimPol is a monomeric enzyme belonging to the archaeal-eukaryotic primase (AEP) superfamily (16). Its catalytic core contains three highly conserved motifs (see Figure 1A), which build the dNTP binding site used for elongation (17). To start primer synthesis, PrimPol requires a unique zinc finger (ZnF)-containing C-terminal domain, which facilitates binding of the first 5′-nucleotide (6,18). Beside its function as a primase, PrimPol behaves *in vitro* as a polymerase with very low processivity (9,19), suggesting the need for a cofactor to function optimally in the cell. In contrast to some other TLS polymerases (20), PrimPol's activity is not regulated by the sliding clamp Proliferating Cell Nuclear Antigen (PCNA) (21), suggesting that another factor might control its function. Indeed, polymerase δ-interacting protein 2 (PolDIP2; also known as PDIP38) and single stranded DNA binding protein Replication Protein A (RPA) (22–24) are able to stimulate PrimPol-dependent DNA synthesis. The PolDIP2-dependent mechanism of PrimPol polymerization stimulation has however not yet been fully clarified.

Here, we show that PrimPol uses a flexible loop located near its AEP catalytic core to interact with the co nserved ApaG-like C-terminal region of PolDIP2 (25), which is essential for the stimulation of its primer extension activity. Using a structural model of PolDIP2 ApaG-like domain, we identify a unique arginine cluster that we show is critical for PrimPol stimulation. Moreover, nucleotide binding assays show that these arginine residues are able to assist dNTP recruitment to the PrimPol-PolDIP2 complex. Taken together, our data shows that PolDIP2 stimulates the processivity of PrimPol by increasing the affinity for the incoming dNTPs and that this processivity stimulation mechanism might be critical for the *in vivo* function of PrimPol in DNA damage tolerance.

## MATERIALS AND METHODS

### Purification of recombinant proteins

PrimPol Δ56 (lacking AA 203–258),constructed by the megaprimer method using two asymmetrical PCR before the final amplification, and WT PrimPol were expressed and purified as previously described (9,18,19). For purification of full-length (FL) human PolDIP2 tagged with C-terminal hexa-histidine, a DNA fragment encoding FL-PolDIP2 was cloned into pET21d (+) plasmid. This construct was also used for the preparation of expression vectors and purification of all other truncated or mutated PolDIP2 protein variants, except for T1-PolDIP2. Plasmids coding for FL-PolDIP2 and T2-PolDIP2 were transformed in *E. coli* ArcticExpress (DE3) cells. An overnight culture was grown at 30°C, and used for inoculation of 1.5 L LB medium that was incubated at 30°C until A_600_ 0.8. The temperature was then reduced to 12°C and protein expression induced by addition of 0.4 mM IPTG. After 18 h of incubation, cells were collected by centrifugation at 4000 × g for 15 min at 4°C. Pellets were washed with ice-cold PBS and then resuspended in lysis buffer A (40 mM Tris–HCl pH 7.5, 5% glycerol, 500 mM NaCl, 1 mM DTT, 0.5% Tween 20, 1 μM Pepstatin, 0.1 mM AEBSF, 1 mM DTT and 1 mM E64). Cells were lysed by pulse-sonication of cell suspensions on ice, and cleared by centrifugation at 25 000 g for 30 min at 4°C. Cell lysates were supplemented with 10 mM imidazole and incubated with Ni-Sepharose excel resin (GE Healthcare, Chicago, IL, USA) for 2 h at 4°C. The beads were washed with buffer B (40 mM Tris–HCl pH 7.5, 5% glycerol, 500 mM NaCl) with 20 mM imidazole, buffer B with 50 mM imidazole, and the proteins were eluted with buffer B containing 500 mM imidazole. Elution fractions containing PolDIP2 were concentrated using a Vivaspin 2, 30 kDa column, and further purified by gel filtration using Superdex 200 Increase 10/300 GL column with buffer C (40 mM Tris–HCl pH 7.5, 5% glycerol, 100 mM NaCl). Protein samples were frozen in liquid N_2_ and stored at −80°C.

For the purification of recombinant T3-PolDIP2, the expression, cell lysis and protein binding to Ni-Sepharose excel resin was performed as described for FL-PolDIP2. Before elution from the resin, bacterial chaperons were removed by incubating the protein in buffer C with 5 mM ATP and 5 mM MgCl_2_. Eluted fractions containing T3-PolDIP2 were loaded on a SP Sepharose High Performance column (GE Healthcare), using buffer D (40 mM Tris–HCl pH 7.5, 5% glycerol, 25 mM NaCl) and eluted in buffer C. To avoid aggregation, eluted fractions were diluted with the same volume of buffer C with 80% glycerol and stored at −20°C. As for purification of T1-PolDIP2, a DNA fragment with GST tag and TEV protease recognition site was introduced at the N-terminal region of T1-PolDIP2. The cell lysate was incubated with Glutathione Sepharose 4 Fast Flow resin for 18 h at 4°C. The resin was washed with buffer B, and the protein was eluted with buffer B containing 40 mM reduced Glutathione (Duchefa Biochemie, Haarlem, The Netherlands). After removal of the nGST tag with TEV protease, fractions containing T1-PolDIP2 were supplemented with 10 mM imidazole and incubated with Ni-Sepharose excel resin, washed with buffer B containing first 20 mM, and then 50 mM imidazole. T1-PolDIP2 was eluted with buffer B containing 500 mM imidazole. Protein samples were frozen in liquid N_2_ and stored at −80°C.

### Oligonucleotides

For DNA replication assays, kinetic studies, and electrophoretic mobility shift assays (EMSA) in Figures 1G, H, and Supplementary Figure S3A–C, a 5′-TET fluorophore-labeled 25-mer oligonucleotide primer (5′-TET-ATAGGGGTATGCCTACTTCCAACTC-3′) was hybridized to a 70-mer oligonucleotide template (5′-GAGGGGTATGTGATGGGAGGGCTAGGATATGAGGTGAGTT**X**AGTGGAGTTGGAAGTAGGCATACCCCTAT-3′, where X was either ‘G’ or ‘8-oxo-G’). In filter binding assays in Figure 5, a non-labeled 25-mer primer (5′-ATAGGGGTATGCCTACTTCCAACTC-3′) was hybridized to the 70-mer oligonucleotide template. For EMSA in Supplementary Figure S3D, the 5′-end of 60-mer ‘GTCC’ oligonucleotide (5′-T_36_CCTGT_20_-3′) was labeled with [γ-^32^P]ATP.

### Primer extension assay

For the PrimPol DNA synthesis, the reaction mixture (10 μl) contained 10 mM Bis-Tris propane (pH 7.0), 40 mM NaCl, 10 mM MgCl_2_, 1 mM DTT, 200 μM dNTPs, 15 nM of hybridized 5′-TET-primer/template DNA, indicated amounts of purified PrimPol (WT or Δ56 mutant) and PolDIP2 (WT, truncated proteins, or mutants). After incubation for 10 min at 37°C, reactions were stopped by addition of 10 μl of formamide loading buffer (0.5% SDS, 25 mM EDTA, 95% v/v formamide and xylene-cyanol). For single hit experiments shown in Figure 1F and Supplementary Figure S2, the reaction composition was modified to include 50 nM hybridized primer/template DNA, 5 nM PrimPol WT, and indicated amounts of PolDIP2 fragments. Those reaction mixtures were incubated at 37°C for 30 min. DNA products of primer extension assays were separated on 10% polyacrylamide gels containing 7 M urea, and directly imaged using a Typhoon 9400 scanner (Amersham Bioscience). The images were quantified with ImageQuant TL 8.1 software (GE healthcare). The percentage of primer that was extended for >3 nt, >10 nt, or was fully extended, was calculated as the ratio between the signal of corresponding products and the signal of the 25-nt primer in the control reaction without protein. When used, the polymerisation termination probabilities at specific nucleotides were calculated as previously described (26).

### Steady-state kinetic analysis of polymerase activity

Single-nucleotide incorporation experiments were performed using 1500 nM primer/template DNA (a mix of 15 nM TET-labeled and 1485 nM unlabeled DNA), with dG as the first templating base. The reaction mixture (10 μL) contained 10 mM Bis–Tris propane (pH 7.0), 40 mM NaCl, 10 mM MgCl_2_, 1 mM DTT, 150 nM of purified PrimPol WT, and 300 nM FL-PolDIP2 WT or 3R>A where indicated. dCTP concentrations from 125 to 5000 μM were used. The reactions were started by the addition of dCTP/Mg^2+^ mix to the other components. Reactions were stopped after 1 or 2 min by addition of 10 μl of formamide loading buffer (0.5% SDS, 25 mM EDTA, 95% v/v formamide and xylene–cyanol). DNA products of primer extension assays were separated on 12% polyacrylamide gels containing 8 M urea and 25% formamide. The gel was imaged and quantified as described for primer extension assays. The initial rate of the reaction at different dCTP concentrations was calculated by fitting the data using a linear fit in Microsoft Excel software. The resulting slopes were plotted against starting dCTP concentrations and fit using the Michaelis-Menten model (OriginPro, OriginLabs), to obtain the *k*_cat_ (turnover number) and *K*_M_ (Michaelis constant) values. The results are an average of two replicates.

**Table 1.** Kinetic parameters of PrimPol alone or in the presence of wild-type or arginine cluster mutant FL-PolDIP2. Single nucleotide incorporation assays were performed on a 25/70mer primer-template substrate, with dG as the first template base and dCTP as the incoming nucleotide. *k*_cat_ – turnover number, *K*_M_ – Michaelis constant for dNTPs. The standard error of the fit is reported for each value

	*k*_cat_ (min^−1^)	*K*_M_ (mM)	*k*_cat_/*K*_M_ (min^−1^ mM^−1^)
PrimPol	0.84 ± 0.02	1.0 ± 0.1	0.84
PrimPol + WT PolDIP2	0.80 ± 0.01	0.16 ± 0.01	5.0
PrimPol + 3R>A PolDIP2	0.90 ± 0.01	0.46 ± 0.3	2.0

### Electrophoretic mobility shift assays (EMSA)

In the experiment presented in Figure 1G, the reaction mixture (10 μl) contained 10 mM Bis-Tris propane (pH 7.0), 10 mM MgCl_2_, 1 mM DTT, 15 nM of hybridized 5′-TET-primer/template DNA and indicated amounts of purified PolDIP2 fragments (FL, T1, T2 or T3). After incubation for 5 min at 37°C, DNA-protein complexes were separated by 10% polyacrylamide gel in Tris-borate buffer, and imaged using a Typhoon 9400 scanner. The percentage of bound DNA was calculated as the ratio between the signal of bound DNA and the signal of the DNA template in the control reaction without protein.

In the experiments presented in Figure 1H, Supplementary Figure S3A, S3C, and S4D, the reaction mixture (10 μl) contained 10 mM Bis–Tris propane (pH 7.0), 300 mM NaCl, 1 mM CaCl_2_, 1 mM DTT, 5 μM dCTP, 15 nM of hybridized 5′-TET-primer/template DNA, and indicated amounts of PrimPol and PolDIP2. After 5 min incubation at 37°C, DNA–protein complexes were separated by 6% polyacrylamide gel in Tris–borate buffer at 4°C, and imaged using a Typhoon 9400 scanner. In the experiment presented in Supplementary Figure S3D, the reaction mixture contained 50 mM Tris–HCl [pH 7.5], 50 mM NaCl, 1 mM MnCl_2_, 1 mM DTT, 2.5% glycerol, 0.1 mg/ml BSA and 2.5 nM polyethylenglycol 4000, 5 nM 5′-[γ-^32^P]-labeled 60-mer ‘GTCC’ oligonucleotide, and indicated concentrations of PrimPol or FL-PolDIP2. After 20 min incubation at 25°C, DNA–protein complexes were separated by 6% polyacrylamide gel in 1× Tris–glycine buffer at 4°C. The gels were then dried and imaged by autoradiography using a Typhoon 9400 scanner.

### Filter-based PrimPol-dNTP binding assay

The reaction mixture (10 μl) contained 10 mM Bis–Tris propane (pH 7.0), 10 mM CaCl_2_, 1 mM DTT, 1 μM dCTP including [α-^32^P]dCTP, 300 nM of hybridized primer/template DNA and 600 nM of purified human PrimPol WT and/or PolDIP2-FL (WT or mutants). After incubation for 5 min at 37°C, the reaction mixture was loaded on a nitrocellulose membrane (0.45 μm; Amersham), and washed with 1 ml of washing buffer (10 mM Bis–Tris propane (pH 7.0), 10 mM CaCl_2_ and 1 mM DTT). The membrane was transferred to a tube and the protein-bound [α-^32^P]dCTP was quantified in a Beckman Coulter LS 6500 Liquid Scintillation Counter using OptiPhase HiSafe 3 (PerkinElmer, Waltham, MA, USA) as solvent.

### Structural comparisons and computational simulation of PolDIP2

Multiple sequence alignments of different PrimPol species, or human PolDIP2 versus human Fbxo3 and bacterial ApaGs, were performed using COBALT (constraint-based multiple alignment tool (27,28) from the National Center for Biotechnology Information (NCBI). The homology models of T3-PolDIP2 (amino acids (AA) 217–368) WT, 3R>A, and R282A were constructed based on the crystal structure of *Shewanella oneidensis* ApaG protein (PDB-ID 1TZA), using I-TASSER web service. Three-dimensional images and surface electrostatic potential calculation were carried out using the Swiss-PdbViewer online server application (http://www.expasy.org/spdbv/) (29).

### Circular dichroism (CD) spectroscopy

CD analyses of PolDIP2 WT and the mutants in Figure 3I and Supplementary Figure S6D were performed on a JASCO J-810 spectropolarimeter (JASCO, Easton, MD, USA) equipped with a Peltier temperature control unit that maintained the temperature at 10°C. A 1 mm quartz cuvette (Hellma, Müllheim, Germany) was used. Before measurements, protein samples were buffer exchanged into a buffer containing 15 mM Tris–HCl (pH 7.5) and 400 mM NaF, with a final protein concentration of ≈5 uM. The measurements shown are the average of five replicates with buffer spectrum subtracted. The plots were prepared in OriginPro (OriginLabs) and were smoothed using the Savitsky–Golay filter. The mean residue ellipticity was calculated using the protein molar concentration and the number of peptide bonds.

## RESULTS

### The C-terminal region of PolDIP2 increases the processivity of PrimPol

An earlier study showed that human PolDIP2 increases the DNA polymerase activity of PrimPol (22). The crosslinking experiments in that study identified several regions as potential PrimPol-PolDIP2 interaction sites and suggested increased PrimPol binding to DNA was the reason for the observed stimulation. To understand in detail how this PrimPol stimulation is mechanistically achieved, we purified four cHis6-tagged PolDIP2 fragments (Figure 1A and B). Apart from the FL-PolDIP2 protein (AA 1–368), we purified an N-terminal T1 fragment (AA 1–50) which covers the mitochondrial targeting sequence (MTS), a middle T2 fragment (AA 51–216) which contains the YccV-like domain, and a C-terminal T3 fragment (AA 217–368), which contains an ApaG-like domain (Figure 1A and B) (25). In addition to representing distinct functional parts of the protein, the fragments also include the three different PrimPol-interaction regions within PolDIP2 (Figure 1A) (22).

To determine if any of these isolated domains of PolDIP2 could sustain the stimulation of PrimPol DNA polymerization, we performed primer-extension assays on a 25/70-mer DNA primer/template substrate in the presence of increasing concentration of each PolDIP2 variant (Figure 1C). PrimPol alone showed limited activity under the set reaction conditions, with the 25 nt 5′-labeled primer extended by 1–3 nt (Figure 1C, lane 2). Addition of FL-PolDIP2 stimulated DNA synthesis by PrimPol, resulting in long DNA products (46% of the DNA substrate was extended further than 3 nt in the presence of 500 nM FL-PolDIP2; Figure 1D), which included run-off products 70 nt in length (Figure 1C, lanes 3–5; Figure 1E). This result is consistent with previous work and confirms that FL-PolDIP2 is able to stimulate PrimPol during DNA synthesis (22). Primer-extension reactions in the presence of the PolDIP2 fragments showed the PrimPol stimulation is mainly dependent on fragment T3 (Figure 1C, lanes 15–17) and to a lesser extent on T1 (Figure 1C, lanes 7–9), but not on T2 (Figure 1C, lanes 11–13). Even the lowest concentration of T3-PolDIP2 fragment (150 nM; Figure 1C, lane 15) showed a robust stimulation of DNA synthesis. Nonetheless, a small reduction of the >28 nt DNA products was detected when compared to the reaction with FL-PolDIP2 (Figure 1C, compare lanes 5 and 17; 32% T3 versus 46% FL in Figure 1D), and fully extended DNA products (68–70 nt) were not accumulated in the presence of T3 at any concentration (Figure 1C and E). Interestingly, addition of 300–500 nM T1-PolDIP2 also showed a modest increase in PrimPol DNA synthesis (Figure 1C, compare lane 2 with lanes 8 and 9; Figure 1D), but this T1 stimulation was substantially weaker compared to the effect of T3- or FL-PolDIP2 (Figure 1C, compare lanes 7–9 with lane 15–17; Figure 1D).

We also purified the putative mitochondrial variant of the protein (AA 51–368), comprising both domains T2 and T3 (Supplementary Figure S1A and S1B). Consistent with the previous study (22), this mitochondrial variant of PolDIP2 (fragments T2 + T3) was unable to stimulate PrimPol DNA synthesis (Supplementary Figure S1C). The mitochondrial PolDIP2 variant's inability to enhance DNA polymerase processivity is most likely the result of T3 fragment inhibition by the T2 domain, since the separate addition of T2 PolDIP2 does not block PrimPol DNA synthesis, but does impede the T3 PolDIP2-dependent PrimPol stimulation (Supplementary Figure S1D).

To test whether PolDIP2 affects DNA product length by increasing PrimPol re-loading efficiency, we performed primer-extension assays with a large excess of template-primer over DNA polymerase (Figure 1F and Supplementary Figure S2). In these conditions, once a primer is extended, the probability that it will be used a second time after dissociation is negligible, and the elongated products therefore derive from a single cycle of synthesis. As observed in standard reaction conditions, both FL- and T3-PolDIP2 considerably increased the length of products, confirming the observed stimulation comes from enhanced processivity and not PrimPol re-loading (Supplementary Figure S2). Additionally, band intensities were measured to calculate termination probability at specific nucleotide positions, as previously described (26). The presence of both FL- and T3-PolDIP2 decreased the termination probability of PrimPol synthesis at position +2; instead, longer products were also observed (Figure 1F, positions +3 to +8, and Supplementary Figure S2), indicating increased processivity of PrimPol during DNA polymerization.

At lower protein concentrations (<100 nM) PolDIP2 has no ability to bind the DNA substrate (22). However, our EMSA experiments showed that at higher concentrations (>150 nM) FL-PolDIP2 is able to bind to the primer-template substrate used in the primer extension assays (Figure 1G, lanes 3 and 4), but that separate fragments (T1, T2 and T3) show little or no DNA binding (Figure 1G, lanes 5–10). PrimPol has no detectable stable binding to the primer-template substrate by itself (Figure 1H); however, FL-PolDIP2 is able to enhance primer-template DNA binding affinity of PrimPol, as demonstrated by the appearance of PrimPol-dependent super-shift band in the presence of the two proteins (Figure 1H-J, Supplementary Figure S3A and S3B). On the contrary, T3-PolDIP2 was unable to alter PrimPol's template-primer binding (Supplementary Figure S3C). Thus, the stimulation of the polymerase activity of PrimPol by the T3 fragment is independent of the intrinsic DNA binding capacity. PrimPol and FL-PolDIP2 are both able to bind to PrimPol's preferred 60-mer GTCC ssDNA substrate, but we were unable to demonstrate a collaboration of the two proteins in ssDNA binding (Supplementary Figure S3D). This might indicate that FL-PolDIP2 stabilizes the binding of PrimPol to the 3′end of the primer, but does not alter its general affinity for the DNA template.

In conclusion, whereas the interaction of PrimPol with FL-PolDIP2 stabilizes the binding of PrimPol to a primer-template substrate, thus explaining an increase in processive polymerization, the stimulation in processivity observed with the T3 fragment of PolDIP2 does not correlate with an improvement in DNA binding.

### A flexible loop in the PrimPol DNA polymerase core is essential for PolDIP2-dependent stimulation

**Figure 2.**
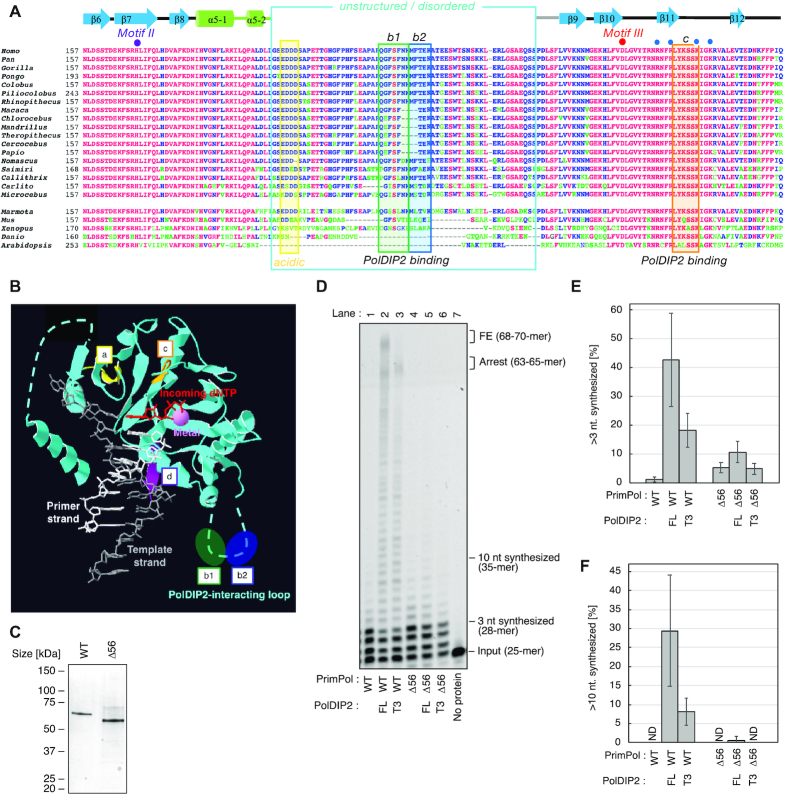
PrimPol AA 203–258 are required for PolDIP2-depended stimulation of DNA synthesis. (**A**) Multiple amino acid sequence alignment of different PrimPol species, in the region spanning AEP motifs II and III. PolDIP2 interacting regions ‘b1’, ‘b2’ and ‘c’ are framed in orange. The unstructured/disordered loop region, deleted in the Δ56 (AA 203–258) PrimPol mutant, is indicated in cyan. The secondary structure elements of human PrimPol (beta-strands and alpha-helices) are indicated above the human sequence. Individual residues involved in dNTP binding (purple dot), catalysis (red dot) or phosphate binding (blue dots) are indicated. The references of the aligned sequences are: *Homo sapiens* (NP_689896); *Pan troglodytes* (XP_001162592); *Gorilla gorilla* (XP_004040738); *Pongo abelii* (XP_024101228); *Colobus angolensis* (XP_011814937); *Piliocolobus tephrosceles* (XP_023076725); *Rhinopithecus roxellana* (XP_010376227); *Macaca fascicularis* (XP_005556457); *Chlorocebus sabaeus* (XP_007998585); *Mandrillus leucophaeus* (XP_011855552); *Theropithecus gelada* (XP_025240422); *Cercocebus atys* (XP_011941669); *Papio anubis* (XP_003899457); *Nomascus leucogenys* (XP_003271526); *Saimiri boliviensis* (XP_010342695); *Callithrix jaccus* (XP_008990643); *Carlito syrichta* (XP_008055415); *Microcebus murinus* (XP_012640401); *Marmota marmota* (XP_015341649); *Mus musculus* (XP_017168376); *Xenopus laevis* (XP_018086024); *Danio rerio* (NP_001032455); *Arabidopsis thaliana* (NP_001154775). (**B**) The reported human PrimPol structure, with a primer/template DNA, metal and incoming dNTP (17), has a non-structured flexible loop (containing sites ‘b1’ and ‘b2’), in addition to sites ‘a’, ‘c’ and ‘d’, that interact with PolDIP2 (22). (**C**) Purified WT PrimPol or Δ56 PrimPol mutant (300 ng) on SDS-PAGE gel stained with InstantBlue. (**D**) Primer extension reactions using 150 nM of PrimPol WT or Δ56 incubated with equimolar amounts (150 nM) of FL-PolDIP2 or T3-PolDIP2, as indicated. These experiments were performed twice, and the percentage of (**E**) >3 nt and (**F**) >10 nt synthesized DNA products was quantified. The average values with standard deviations are shown.

As shown here, the most important PolDIP2 fragment for enhancing PrimPol processivity is T3. Within this fragment, a region here termed ‘C’ (AA 215–223), has been previously shown to directly interact with region ‘b1’ (AA 226–232) in PrimPol (Figures 1A, 2A and B) (22). These PrimPol residues are located within a non-structured, perhaps flexible loop (17), spanning AA 201–260 of human PrimPol (Figure 2A and B). This disordered region is relatively conserved in primates, but largely diverged in PrimPols from other vertebrates and plants; however, the signature of the PolDIP2 interacting region ‘b1’ is still present in PrimPols from rodents and amphibians (Figure 2A).

To investigate if this PrimPol-PolDIP2 interaction is required for PrimPol stimulation, we purified a PrimPol variant with deleted flexible loop, Δ56 PrimPol (lacking AA 203–258; Figure 2C). PrimPol WT and Δ56 variants have comparable primer extension activities, showing that the flexible loop is dispensable for DNA polymerization activity of PrimPol (Figure 2D, compare lanes 1 and 4). However, only the WT PrimPol was efficiently stimulated by FL- and T3-PolDIP2 (Figure 2D, lanes 2–3; quantitative analysis in Figure 2E), highlighting the importance of the deleted region. Addition of FL-PolDIP2 showed a minimal stimulation of Δ56 PrimPol (Figure 2D, compare lanes 4 and 5; Supplementary Figure S4A–C), as judged by quantitation of both short (Figure 2E) and long (Figure 2F) extension products. On the other hand, the T3-PolDIP2 fragment was completely unable to stimulate the Δ56 PrimPol variant (Figure 2D, lane 6; Figure 2E and F), even when higher concentrations of the T3 variant were used (Supplementary Figure S4A–C). FL-PolDIP2 was unable to alter the binding of the template-primer DNA by Δ56 PrimPol, showing that PolDIP2 interaction with AA 201–260 is also required to enhance PrimPol primer-template affinity (Supplementary Figure S4D).

In conclusion, the flexible loop in PrimPol (AA 201–260), which contains a binding site for the interaction with the T3 region, is essential for PolDIP2-dependent stimulation of PrimPol DNA polymerization.

### PolDIP2 contains a putative pyrophosphate binding motif and an Arginine cluster

**Figure 3.**
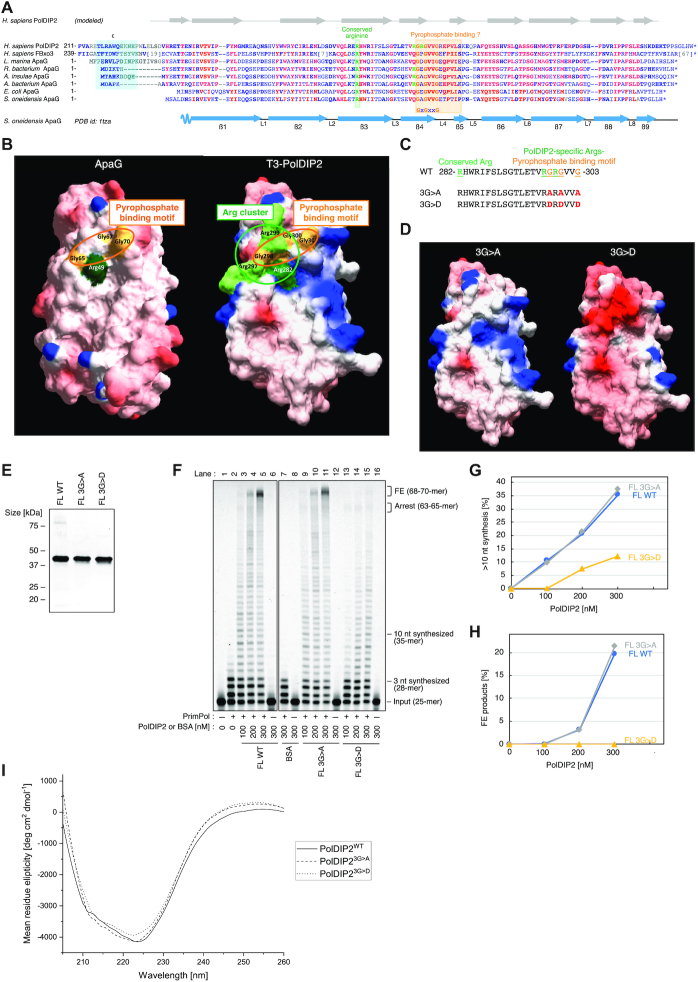
The conserved PolDIP2 pyrophosphate binding motif is dispensable for PrimPol stimulation. (**A**) The T3 domain of human PolDIP2 (AA 217–368) has homology to FBxo3 and ApaG proteins, and contains a pyrophosphate binding motif and a proximal Arg-cluster. The amino acid sequences aligned were: *Homo sapiens* PolDIP2 (NP_056399); *Homo sapiens* FBxo3 (XP_011518282.1); *Labrenzia marina* ApaG (POF34899.1); *Rhodospirillaceae bacterium* ApaG (HBC05999.1); *Aestuariispira insulae* ApaG (RED46168.1); *Alphaproteobacteria bacterium* ApaG (OLB70390.1); *Escherichia coli* ApaG (ART45711.1); *Shewanella oneidensis MR-1* ApaG (AAN56626). Region ‘C’ in PolDIP2, involved in interaction with the ‘b1’ region of PrimPol is indicated with a cyan box. The positions of the pyrophosphate binding motif (boxed in orange), G^298^xG^300^xxG^303^ in human PolDIP2, and the Arg-cluster residues Arg^282^, Arg^297^, and Arg^299^ (green letters) are indicated. Residues with the highest conservation are indicated in red, and other conserved residues are indicated in bold blue letters. The secondary structure elements (beta-strands β1 to β9 and loops L1 to L8) corresponding to *S. oneidensis* MR-1 ApaG (PDB id: 1TZA) is indicated below the multiple alignment. The secondary structure elements of the predicted model for the T3 domain of *H. sapiens* PolDIP2 are indicated above the multiple alignment. (**B**) Left: surface electrostatic potential of the *S. oneidensis* MR-1 ApaG (PDB id 1TZA), Right: surface electrostatic potential of the 3D-structure model for T3-PolDIP2, obtained using the I-TASSER web tool with PDB-ID 1TZA as a template. The residues of the Arg-cluster and pyrophosphate binding motif are shown with green and orange, respectively. The amino acid sequence (**C**) and the structural models (**D**) of PolDIP2 pyrophosphate binding motif mutants used in this study. Two triple mutations 3G>A (G298A/G300A/G303A) and 3G>D (G298D/G300D/G303D) were introduced into FL-PolDIP2. (**E**) Purified FL-PolDIP2 WT or mutants (3G>A or 3G>D) (300 ng) on SDS-PAGE gel stained with InstantBlue. (**F**) Primer extension experiments using 150 nM of WT PrimPol in the presence of the indicated amounts of FL-PolDIP2 WT, 3G>A, or 3G>D. The ratio of DNA products extended for >10 nt (**G**), or fully extended (**H**). (**I**) Circular dichroism spectra of FL-PolDIP2 WT and the pyrophosphate binding domain mutants 3G>A and 3G>D.

Sequence comparison shows that the T3-PolDIP2 fragment contains a bacterial protein ApaG-like domain that also shows sequence similarity with the human F-Box only 3 (FBxo3) protein. A multiple amino acid sequence alignment of the T3 domain of human PolDIP2 with human FBxo3 and the closest ApaGs from various species confirmed the structural similarity (Figure 3A). Strikingly, some of these aligned sequences also contain the region ‘C’ of PolDIP2, involved in interaction with PrimPol (cyan box in Figure 3A) (22). More importantly, one of the most conserved portions of this alignment, present in all three types of proteins, shares a loop with a highly conserved motif (GxGxxG), that has been proposed to be involved in binding to the pyrophosphate moiety of a nucleotide derivative (30,31). The crystal structure of *S. oneidensis* ApaG (PDB ID: 1TZA; doi: 10.2210/pdb1tza/pdb) shows that the putative pyrophosphate binding motif (G^65^xG^67^xxG^70^) forms a double turn between two beta strands (β4 and β5; see Figure 3A), building a wall at the end of a channel that serves as a cavity to bind pyrophosphate (Figure 3B, left). At the bottom of this channel, a specific arginine (Arg^49^) appears to be the likely candidate to interact with the phosphate moiety of nucleotide derivatives. As the amino acid sequence identity between T3-PolDIP2 and *S. oneidensis* ApaG was about 30%, high enough to generate a homology model (32), we built a structural model of the T3-PolDIP2 fragment (Figure 3B, right; Supplementary Figure S5A and S5B). Interestingly, the T3 domain of PolDIP2 also contains an apparent binding channel, but with a unique positively-charged Arginine cluster (Arg^282^, Arg^297^, Arg^299^) at the bottom, that partly overlaps with its putative pyrophosphate binding motif (298-GRGVVG) (Figure 3A and B). The first of these Arginine (Arg^282^) residues aligns with Arg^49^ of *S. oneidensis* ApaG, whereas the other two residues (Arg^297^, Arg^299^) are highly conserved in PolDIP2 of higher eukaryotes, and occasionally in some ApaG sequences (Figure 3A).

### The Arginine cluster in PolDIP2 is essential for stimulation of PrimPol DNA synthesis

To investigate if the pyrophosphate binding motif in PolDIP2 has an essential role in PrimPol stimulation, we purified two mutant variants of FL-PolDIP2 with alterations in the G^298^xG^300^xxG^303^ motif: a triple mutant with conservative 3G>A (G298A/G300A/G303A) replacement, and a more radical 3G>D (G298D/G300D/G303D) substitution that would also affect the surrounding electrostatic potential (Figure 3C-E). Our results show that the conservative alteration of the pyrophosphate binding motif did not affect PrimPol stimulation, since the FL-3G>A mutant had a wild-type-like activity, stimulating PrimPol in primer extension assays (Figure 3F, compare lanes 3–5 with lanes 9–11; see quantitation at Figure 3G and H). On the other hand, the Gly-to-Asp triple mutant (FL-3G>D) shows a strong reduction in the level of PrimPol stimulation (Figure 3F, compare lanes 3–5 to lane 13–15), especially evident for primers that are extended by >10 nt (Figure 3G), or as measured by quantification of the full extension products (Figure 3H).

Importantly, both 3G>A and 3G>D substitutions did not considerably affect the overall structure of these proteins, since both showed WT PolDIP2-like circular dichroism spectra (Figure 3I). These results suggest that the pyrophosphate binding motif is not vital for PrimPol stimulation, but on the other hand, the region surrounding the GxGxxG motif is essential, leading us to speculate that the positively charged Arg-cluster might have an important function.

**Figure 4.**
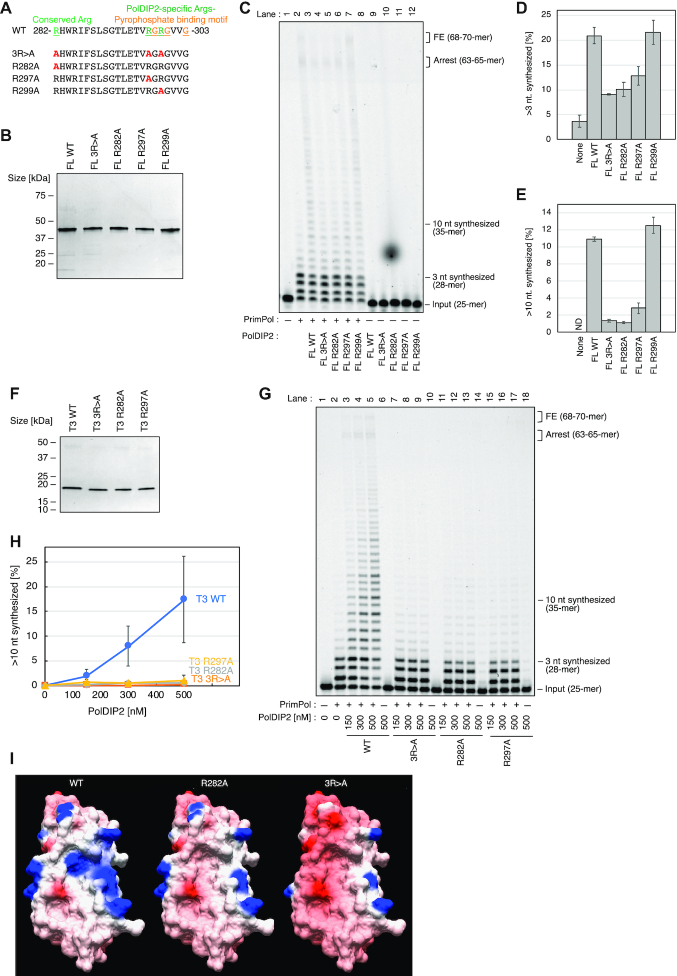
An Arg-cluster in PolDIP2 is required for PrimPol stimulation. (**A**) Amino acid sequences (AA 282–303) of the PolDIP2 Arg-cluster mutants used in this study. A triple mutation 3R>A (R282A/R297A/R299A), and three single Arg alterations (R282A, R297A, or R299A) were introduced into FL- or T3-PolDIP2. (**B**) Purified FL-PolDIP2 WT and mutants (300 ng) were loaded on SDS-PAGE gel and stained with InstantBlue **(C–E)** PrimPol stimulation of DNA synthesis by the Arg-cluster FL-PolDIP2 mutants. (**C**) Primer extension reaction using 150 nM of WT PrimPol protein in the presence of equimolar amounts (150 nM) of the indicated FL-PolDIP2 variants. This experiment was repeated twice, and the percentage of (**D**) >3 nt and (**E**) >10 nt synthesized DNA products was quantified. The average values and absolute errors are shown with error bars. (**F**) Purified T3-PolDIP2 WT and mutants (3R>A, R282A, or R297A) (300 ng) on SDS-PAGE gel stained with InstantBlue. **(G, H)** Stimulation of PrimPol DNA synthesis by the T3-PolDIP2 mutants. (**G**) Primer extension reactions with 150 nM of WT PrimPol, in the presence of the indicated amounts of T3-PolDIP2 variants. This experiment was repeated twice, and the percentage of >10 nt synthesized DNA products was quantified (**H**). The average values with absolute errors are shown. **(I)** The surface electrostatic potential of the T3-PolDIP2 variants (WT-T3, T3-R282A and T3-R282A/R297A/R299A).

To address the function of the PolDIP2 Arg-cluster in PrimPol stimulation, we purified FL-PolDIP2 variants with modifications in the Arg-cluster: a triple mutant R282A/R297A/R299A (FL-3R>A), and the single amino acid residue alterations FL-R282A, FL-R297A and FL-R299A (Figure 4A and B). When tested for their ability to stimulate PrimPol in primer extension assays (Figure 4C), the FL-R299A PolDIP2 mutant displayed a FL-WT PolDIP2-like ability to stimulate PrimPol, showing that the Arg^299^ residue is not critical in this respect (Figure 4C, compare lanes 3 and 7; Figure 4D and E). On the other hand, both other single mutant variants of PolDIP2, FL-R282A and FL-R297A, showed considerably reduced capacity to stimulate PrimPol (Figure 4C, lanes 5 and 6), exposing the importance of these two residues. As expected, the triple mutant 3R>A also showed a similar reduction in the capacity to stimulate PrimPol (Figure 4C, lane 4). In agreement with these results, the structural model of the T3 fragment arginine mutants (R282A and R297A) predicts a strong decrease in the positive surface charge observed in the WT PrimPol T3 fragment structural model (Figure 4I) in the vicinity of the pyrophosphate binding motif.

Together with our observations with mutating the pyrophosphate binding motif, the overall charge in these two overlapping conserved regions appears to be critical for PrimPol stimulation.

We speculated that the remaining PrimPol stimulation activity observed with FL-R282A and FL-R297A PolDIP2 mutants, clearly observed at high PolDIP2 concentrations (Supplementary Figure S6A-D) was a consequence of the moderate ability of the PolDIP2 fragment T1 to stimulate PrimPol (Figure 1C), and not the contribution of the actual T3 domain. To test this, we purified the T3-PolDIP2 fragment variants T3-R282A, T3-R297A and the triple mutant T3-3R>A (Figure 4F). As expected, these mutants did not stimulate PrimPol, whereas the T3-WT PolDIP2 fragment was able to increase PrimPol DNA synthesis (Figure 4G and H). Together, these data show that the PolDIP2 residues Arg^282^ and Arg^297^ are critical for stimulation of DNA synthesis by PrimPol.

### The arginine cluster in PolDIP2 favors dNTP binding by PrimPol

**Figure 5.**
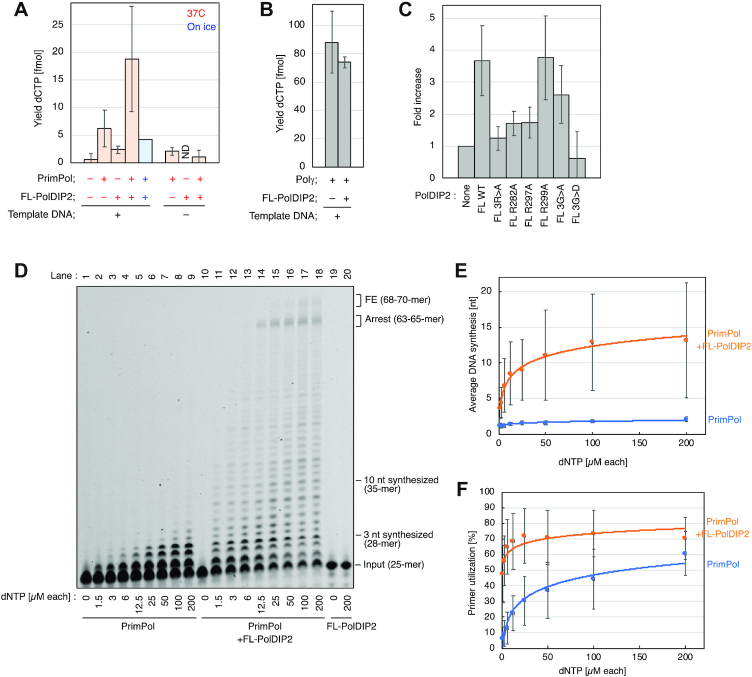
The PolDIP2 Arg-cluster increases PrimPol-dependent dNTP binding, facilitating PrimPol DNA synthesis stimulation at *in vivo* relevant dNTP levels. (**A-C**) Influence of PolDIP2 on the binding of dCTP to either PrimPol or Polγ. (**A**) dNTP binding assay was performed in the presence of 1 μM dCTP including [α-^32^P]dCTP, 300 nM of primer/template DNA, 600 nM of WT PrimPol, and/or equimolar (600 nM) of WT FL-PolDIP2 incubated at 37°C (red bars) or on ice (blue bar) for 5 min in the presence of Ca^2+^. (**B**) dNTP binding as described for Figure 5A, but in the presence of 300 nM PolγA, 450 nM PolγB and 600 nM WT FL-PolDIP2. (**C**) The influence of the PolDIP2 Arg-cluster on dNTP binding efficiency. Conditions were as described for Figure 5A in the presence of 600 nM of the indicated FL-PolDIP2 variants. **(D–F)** PolDIP2-dependent PrimPol stimulation at a variety of dNTP concentrations. (**D**) Primer extension reactions with 150 nM of PrimPol, and 150 nM of FL-PolDIP2 in the presence of indicated dNTP concentrations. The average length of the DNA products (**E**) and the percentage of DNA substrates extended with 1 nt or more (**F**) were quantified. The line is included as a visual aid only.

Despite the identification of critical residues for PrimPol stimulation in PolDIP2 and the PolDIP2-interacting region in PrimPol, it remains unclear what is the mechanism by which T3-PolDIP2 stimulates DNA synthesis by PrimPol. One strategy to enhance the processivity of a DNA polymerase could involve increasing its binding affinity for dNTPs (33). This is particularly relevant for PrimPol, whose low affinity for dNTPs might hinder its ability to synthesize DNA *in vivo* (34,35). To investigate if the presence of PolDIP2 influences dNTP binding by PrimPol, we performed filter binding assays in the presence of a DNA primer/template and the complementary nucleotide for the first position after the primer, [α-^32^P]dCTP. Ca^2+^ was used as the metal cofactor, since it supports PrimPol binding to the incoming dNTP, but not primer extension (Figure 5A) (17,36). As expected, the dNTP binding capacity of PrimPol alone was much lower when compared with Polγ, the replicative mitochondrial DNA polymerase (compare the y-axis scale of Figure 5A and B), and PolDIP2 alone was very inefficient in binding dCTP (Figure 5A). However, when both PrimPol and PolDIP2 were added simultaneously, a 3-fold increase in the amount of bound dCTP was observed when compared to PrimPol alone. At the same time, the dCTP binding of Polγ was unaffected by PolDIP2 addition (Figure 5B), demonstrating the specificity of this interaction. We also observed that PolDIP2 was unable to increase the dCTP binding efficiency on ice (Figure 5A, blue bar).

Next, we investigated if the Arg-cluster in PolDIP2, critical for the stimulation of DNA polymerization activity of PrimPol, is also important for its dNTP binding efficiency. Indeed, the PolDIP2 arginine cluster mutants (FL-R282A, FL-R297A and FL-3R>A), as well as the pyrophosphate binding motif alteration FL-3G>D, only mildly stimulated the dNTP binding (Figure 5C), in line with their inability to stimulate DNA synthesis by PrimPol (Figure 3F and 4G). Conversely, PolDIP2 alterations FL-R299A and FL-3G>A, unaffected in their ability to stimulate PrimPol (Figure 3F and 4C), increased the dNTP binding efficiency (Figure 5C).

If PolDIP2 increases the affinity of PrimPol for nucleotides, that should result in increased DNA polymerase capabilities at lower, *in vivo* relevant dNTPs concentrations (37). To investigate this option, we performed primer extension assays in the presence of a range of dNTP concentrations (Figure 5D). As expected, PrimPol alone poorly elongates the primer beyond nucleotide +1 at dNTP concentrations thought to be physiologically relevant (up to 12.5 μM, Figure 5D, lanes 2–5). However, addition of PolDIP2 results in a substantial amount of DNA fragments extended for more than 3 nucleotides (Figure 5D, lanes 11–14). In fact, The PolDIP2-dependent stimulation of PrimPol synthesis was already evident using the lowest concentration of dNTPs (1.5 μM; Figure 5D, compare lanes 2 and 11; Figure 5E). Interestingly, PrimPol's improved affinity for dNTPs also leads to a strong increase in primer utilization at low dNTP concentrations; the latter was only slightly increased at higher dNTP concentrations (100–200 μM; Figure 5F). To quantitatively describe the observed stimulation, we also performed a kinetical characterization of PrimPol in the presence or absence of PolDIP2. Strikingly, WT PolDIP2 significantly decreased the *K*_M_ of PrimPol for the incoming dNTP, resulting in approximately six times higher catalytic efficiency, *k*_cat_/*K*_M_ (Table 1). On the other hand, the *K*_M_ was less affected when 3R>A PolDIP2 was used together with PrimPol, in-line with the considerably lower stimulation observed in primer extension experiments. Together, these data suggest that PolDIP2 stimulation of PrimPol DNA polymerase activity would be particularly beneficial at physiologically relevant dNTP concentrations.

## DISCUSSION

PolDIP2 has the ability to increase the DNA synthesis efficiency of PrimPol (22), however, previous studies have not addressed in detail the mechanism behind this stimulation. Based on the analysis of protein-protein interaction sites between PrimPol and PolDIP2 (22), we designed several constructs and mutants of these two proteins, that allowed us to identify the regions essential for polymerization stimulation (Figure 1).

As previously reported, only the FL-PolDIP2, but not the mitochondrial variant (Mito-PolDIP2), is able to stimulate the DNA polymerase activity of PrimPol (Figure 1C, Supplementary Figure S1) (22). Because Mito-PolDIP2 lacks fragment T1, we initially suspected that the T1 segment of PolDIP2 (which interacts with three separate regions ‘a’, ‘c’ and ‘d’ in PrimPol) might be essential for PrimPol stimulation. However, as shown in Figure 1C, the T1-PolDIP2 fragment by itself had only a minor ability to stimulate PrimPol; instead, the C-terminal region (T3) of PolDIP2 showed intrinsic and robust PrimPol stimulation. Same was observed in single turnover experiments where both FL-PolDIP2 and T3-PolDIP2 were able to strongly increase the processivity of PrimPol, generating long DNA products (Figure 1F, Supplementary Figure S2). This suggests that the major contribution to PrimPol stimulation stems from the T3 domain of PolDIP2, whereas the T1 region only has a minor impact. Our results are therefore consistent with the conclusion that in Mito-PolDIP2 (i.e. fragments T2 + T3), the fragment T2 prevents fragment T3 to bind PrimPol in a productive way, thus inhibiting the stimulation observed with the T3 domain alone (Supplementary Figure S1D). It is at this point exciting to speculate that, in mitochondria, the Mito-PolDIP2 is unable to stimulate PrimPol. Unless other regulatory factors are able to deter the T2 fragment-dependent inhibition of T3 activity, this could indicate that PrimPol is mainly active as a primase in mitochondria (11,15). However, this hypothesis will need to be tested in future experiments.

PolDIP2 has been proposed to increase the performance of TLS DNA polymerases in DNA damage tolerance (38). In the case of PrimPol, earlier studies suggested that PolDIP2 achieves this by increasing the affinity of PrimPol for DNA (22). FL-PolDIP2 is indeed able to improve the binding of PrimPol to a primer-template DNA substrate (Figure 1H), conceivably by specific stabilization of the PrimPol interaction with the 3′ terminus of the primer. In contrast, the T3-PolDIP2 fragment shows robust PrimPol stimulation (Figure 1C), despite its inability to bind either the primer-template DNA itself (Figure 1G), or to assist PrimPol in binding to it (Supplementary Figure S3C). Whereas binding to DNA probably contributes to the observed stimulation of PrimPol polymerase activity by PolDIP2, particularly in the case of full-length protein, it is not necessarily the sole reason behind the effectiveness of the PolDIP2 in PrimPol stimulation.

Our results are therefore consistent with a hypothesis where PolDIP2, through its T3-domain, interacts with PrimPol and increases its affinity for the incoming dNTP, as indicated by filter binding assays (Figure 5A-C) and kinetic measurements (Table 1). We show that the area around the pyrophosphate binding motif, and two residues of the newly identified arginine cluster, are critical for the observed stimulation of PrimPol polymerase activity, since dismantling of the pyrophosphate binding motif, or alterations in the arginine cluster of PolDIP2 precluded the stimulation of dNTP binding (Figure 5C), and the consequent increase in polymerization by PrimPol (Figure 3 and 4). These findings are also in agreement with the role of the related bacterial ApaG protein, which has been proposed to have the potential to interact with pyrophosphate moiety or even with complete nucleotides (39).

**Figure 6.**
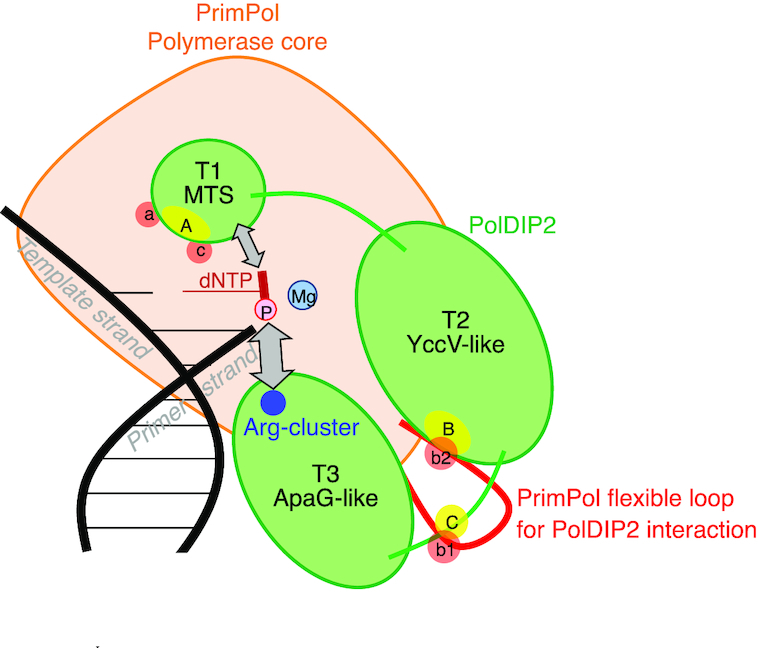
Mechanistic model for PrimPol stimulation by PolDIP2. From both our data presented in the manuscript and the structural insight of PrimPol (Figure 2C), we propose the following model for PolDIP2-dependent PrimPol polymerase stimulation. The PolDIP2 region ‘A’ in the T1 fragment interacts with the PrimPol regions ‘a’ and ‘c’, in the vicinity of the dNTP binding pocket. PolDIP2 region ‘B’ and ‘C’ in the T2 and T3 fragment can interact with PrimPol regions ‘b2’ and ‘b1’, respectively, which locate in the flexible loop of PrimPol (red). These multiple protein-protein interactions can be responsible for the function of the unique PolDIP2 Arg-cluster close to the pyrophosphate binding motif, (both present in T3 fragment), and also the T1 fragment of PolDIP2, likely contributing to optimize dNTP binding at the PrimPol 3′-elongation site, thus favoring processive DNA polymerization. This stimulation is likely crucial to facilitate elongation across DNA lesions.

Contributing to the stimulation is also the T1 domain of PolDIP2, which is able to interact with regions ‘a’ and ‘c’ of PrimPol (Figure 6). Considering that region ‘a’ is involved in the positioning of the templating base, whereas region ‘c’ contains Lys297, a direct ligand of the pyrophosphate moiety of the 3′-incoming nucleotides (17), it is tempting to speculate that the T1 region contributes to the induced-fit step required to select the correct nucleotide.

Considering the inherent low affinity that PrimPol has for dNTPs (34,35), and the presumably low dNTPs concentrations *in vivo* (37) increased affinity for dNTPs could be vital for the *in vivo* functions of PrimPol.

Our work also shows that the stimulation of PrimPol by PolDIP2 requires a direct and very specific interaction between these two proteins (Figure 2), involving the T3 domain of PolDIP2 and PrimPol AA 201–260. The later forms a disordered loop, which is dispensable for intrinsic DNA polymerization, but essential for PolDIP2-dependent stimulation of PrimPol, suggesting it could serve specifically as a scaffold to recruit PolDIP2. Also, no direct contacts with the primer strand were seen in the crystal structure of human PrimPol in ternary complex with DNA and dNTP (17). The interaction of the T3 domain of PolDIP2 with the ‘b1’ region embedded in the flexible loop of PrimPol could promote such a contact, resulting in an improvement of PrimPol′s primer grip. Such a reinforced binding of the primer could enhance processivity, an effect observed in stimulation of PrimPol by PolDIP2. In agreement with this idea, a more robust interaction with the primer/template could indirectly imply an increase in affinity for the correct incoming nucleotides, which is in agreement with our observations (Figure 5A–C and Table 1).

In summary, we show that at physiologically relevant dNTP concentrations (<12.5 μM), PrimPol is a non-processive DNA polymerase, unable to incorporate more than a single nucleotide (Figure 5D), implying that PrimPol by itself is likely inactive *in vivo* as a polymerase. Association with PolDIP2 could activate PrimPol *in vivo* to overcome the relatively poor affinity for dNTPs and provide processivity. Moreover, it is tempting to speculate that PolDIP2 could boost the dNTP binding potential of PrimPol, especially when the insertion has to occur opposite a templating base lesion. Future experiments with DNA-damage-containing DNA substrates could address this intriguing possibility.

## Supplementary Material

gkab049_Supplemental_File
