# Supplementary material for: A unique arginine cluster in PolDIP2 enhances nucleotide binding and DNA synthesis by PrimPol
Source: Nucleic Acids Res. 2021 Feb 3;49(4):2179–91. doi: 10.1093/nar/gkab049 (PMC7913696; doi:10.1093/nar/gkab049)
Supplement: gkab049_Supplemental_File [file gkab049_supplemental_file.pdf]

Supplementary Data

A unique arginine cluster in PolDIP2 enhances nucleotide binding and DNA synthesis by PrimPol.

Kazutoshi Kasho[1], Gorazd Stojkovič[1], Cristina Velázquez-Ruiz[2], Maria Isabel Martínez-Jiménez[2], Mara Doimo[1], Timothée Laurent[1], Andreas Berner[1], Aldo E. Pérez-Rivera[2], Louise Jenninger[3], Luis Blanco[2] and Sjoerd Wanrooij*[1]

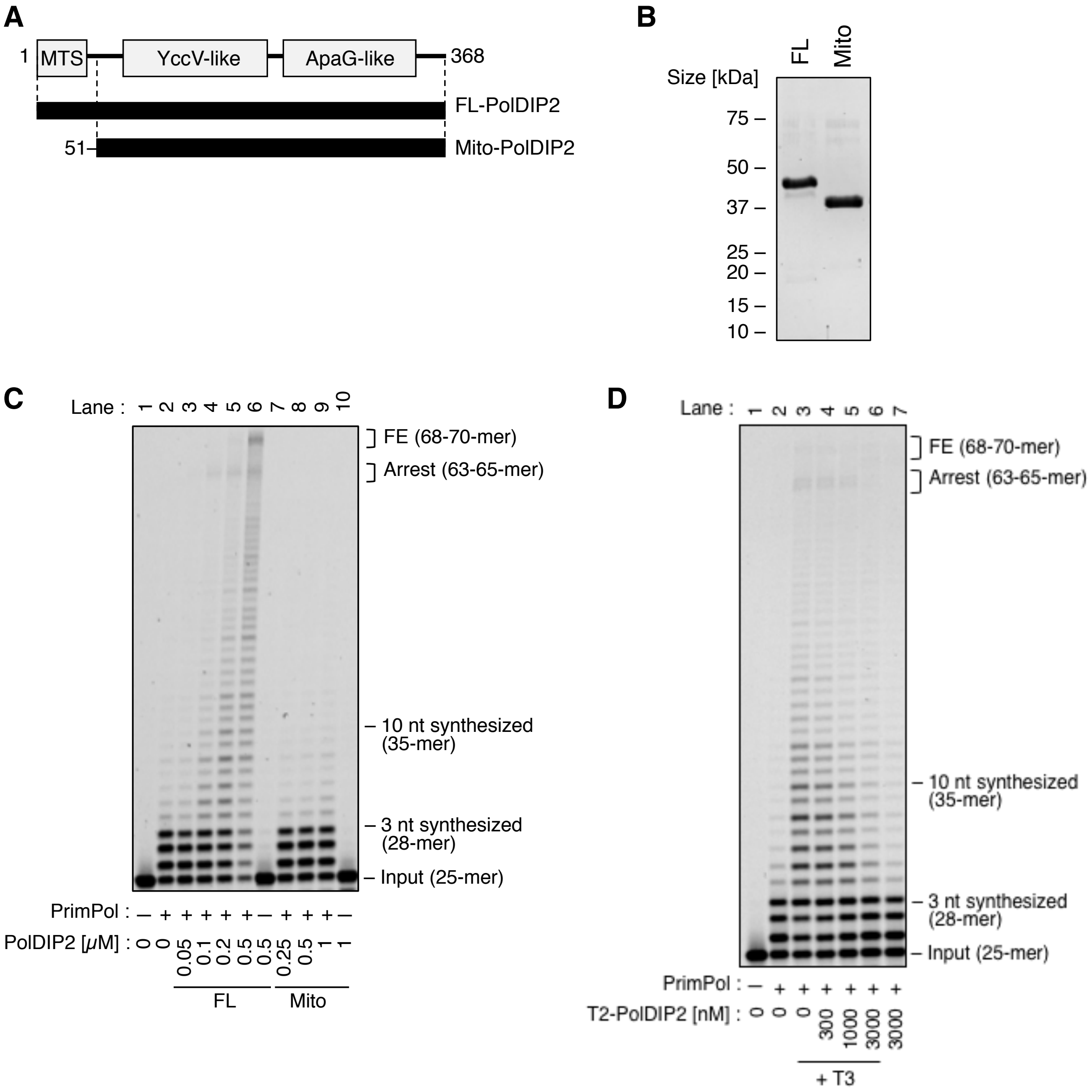

Supplementary Figure S1. Mitochondrial PolDIP2 is unable to stimulate PrimPol in DNA synthesis (A) Schematic presentation of the FL-PolDIP2 and the mitochondrial variant of PolDIP2 (Mito-PolDIP2). (B) Purification of Mito-PolDIP2. 300 ng of each purified PolDIP2 variant on SDS-PAGE gel stained with InstantBlue (Expedeon, UK). (C) PrimPol primer extension assays in the presence of FL- and Mito-PolDIP2. Reactions with 150 nM of PrimPol in the presence of 15 nM of 5'-TET-labeled primer/template DNA and the indicated amount of PolDIP2 variants. (D) PrimPol primer extension assays in the presence of T2 and T3PolDIP2. Reactions with 150 nM of PrimPol in the presence of 15 nM of 5'-TET-labeled primer/template DNA and the indicated amount of T2-PolDIP2 with or without mixing 300 nM T3-PolDIP2, or 300 nM Mito-PolDIP2.

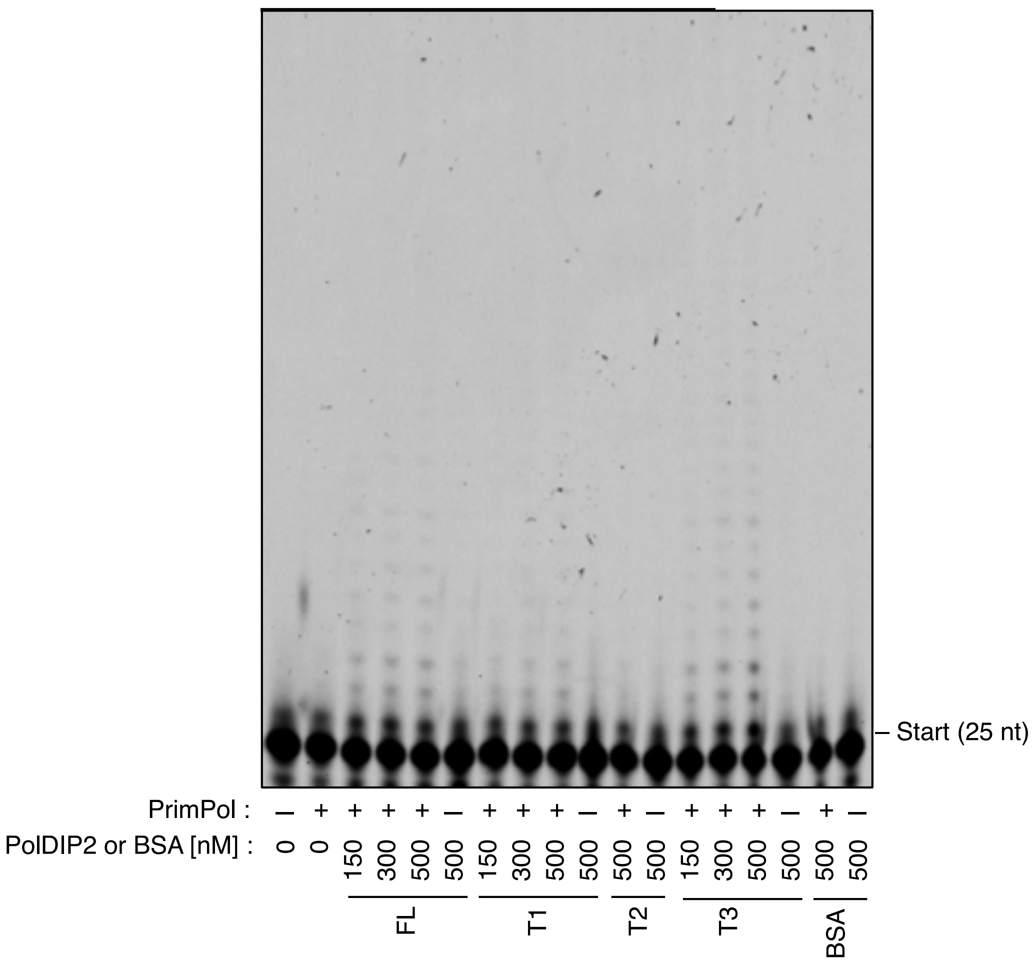

Supplementary Figure S2. PrimPol primer extension experiments in single hit conditions. Polymerization reactions in the presence of primer/template DNA (50 nM), a 10-fold excess molarity over PrimPol (5 nM) to minimize re-initiation of synthesis after a termination event.

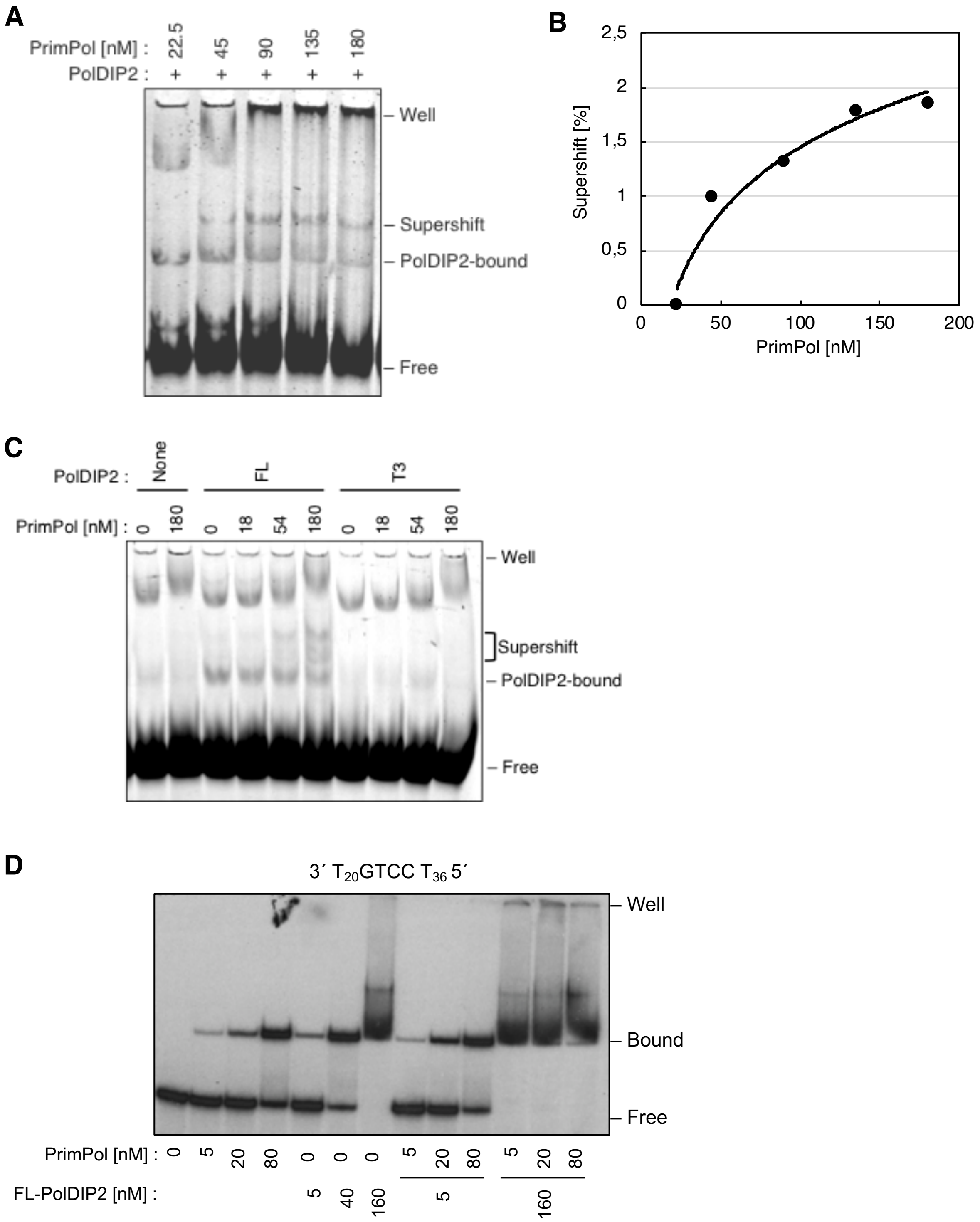

Supplementary Figure S3. Influence of PolDIP2 on PrimPol binding to different DNA templates, related to Figure 1H. (A) Titration of PrimPol. 15 nM of 5'-TET-primer/template DNA was mixed with the indicated concentrations of PrimPol and 300 nM of FL-PolDIP2 and incubated at 37 °C for 5 min. The PrimPol-dependent complexes are indicated with ‘Supershift’. PolDIP2 bound to DNA (‘PolDIP2-bound’), and unbound DNA (‘Free’) are indicated. (B) Quantification of Supplementary Figure

S3A showing the proportion of DNA bound to PolDIP2 and PrimPol-dependent complexes. (C) The T3 PolDIP2 fragment is unable to stimulate PrimPol-DNA binding. 15 nM of 5'-TET-primer/template DNA was mixed with the indicated concentrations of PrimPol and 300 nM of FL- or T3-PolDIP2 variants and incubated at 37 °C for 5 min. (D) Interaction of PrimPol and PolDIP2 with ssDNA containing a favored PrimPol priming site (GTCC). The binding affinity of PrimPol and PolDIP2 were compared by EMSA using the specific 60-mer GTCC template. 5 nM of ^{32}P-labeled GTCC template was mixed with the indicated concentrations of PrimPol and FL-PolDIP2. PrimPol or PolDIP2 bound to DNA (PolDIP2-bound), and unbound DNA (free) are indicated.

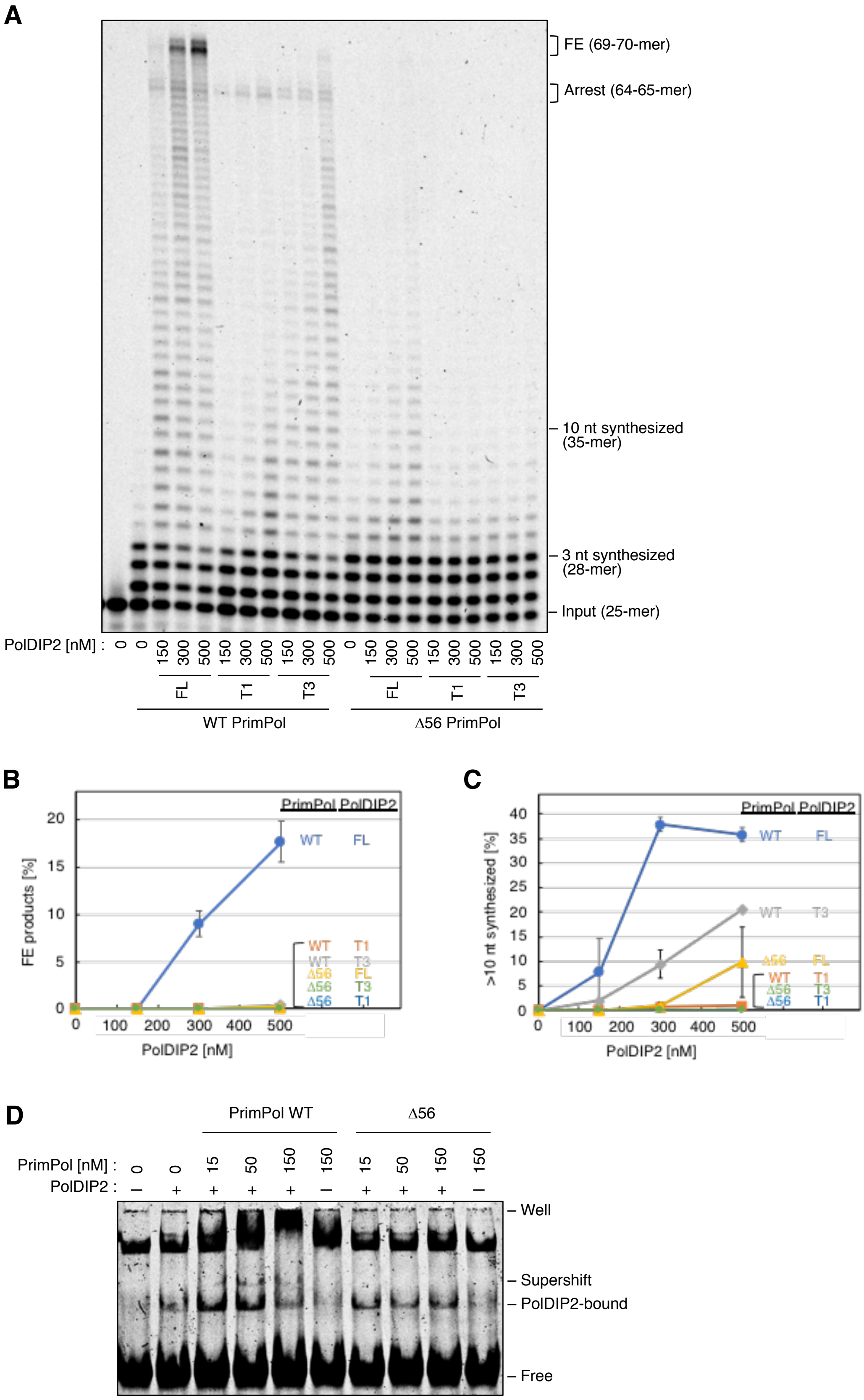

Supplementary Figure S4. AA 203-258 from PrimPol are required for PolDIP2-depended stimulation of DNA synthesis. (A) Primer extension reactions using 15 nM

of 5'-TET-labeled template DNA and 150 nM of WT or Δ56 PrimPol incubated with the indicated amounts of PolDIP2 variants. Quantification of (B) full extension and (C) >10 nt synthesized DNA products. These experiments were performed twice, and averages with standard deviations are shown with error bars. (D) The Δ56 PrimPol mutant is unable to bind to the PolDIP2-DNA complexes. The reaction contained 15 nM of 5'-TET-primer/template DNA and the indicated concentrations of WT or Δ56 PrimPol and 300 nM of FL-PolDIP2. The incubation was done at 37 °C for 5 min.

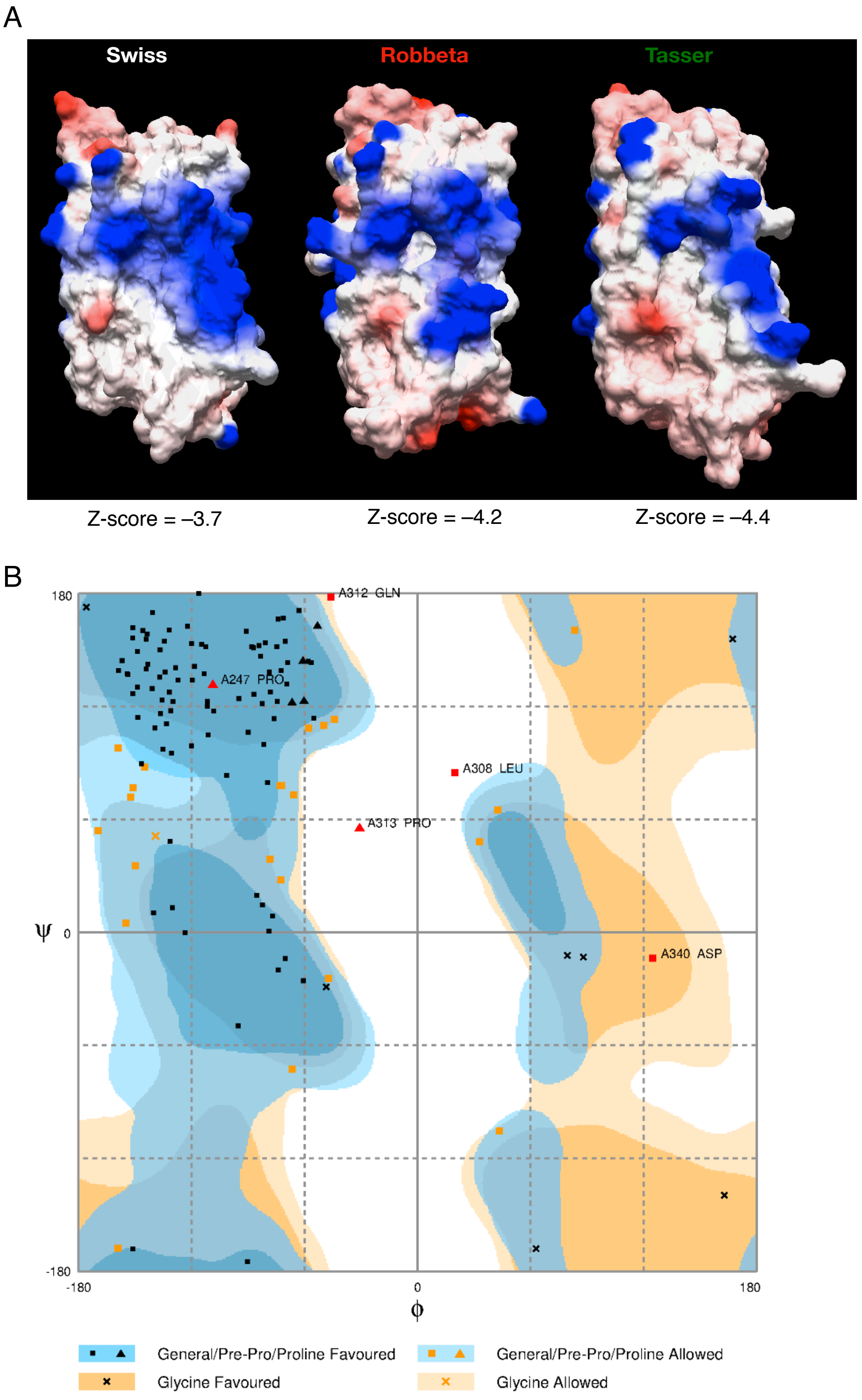

Number of residues in favoured region (~98.0% expected) : 100 (78.1%)
Number of residues in allowed region (~2.0% expected) : 23 (18.0%)
Number of residues in outlier region : 5 (3.9%)

Supplementary Figure S5. Structural model of PolDIP2-T3. (A) A Comparison of the *S. oneidensis* MR-1 ApaG (PDB id: 1TZA)-based modeled structures of PolDIP2-T3

with SWISS-MODEL, Robbeta, and I-TASSER web services, respectively. (B) The amino acid allowance of the constructed PolDIP2-T3 model (I-TASSER) was calculated with Ramachandra Plot.

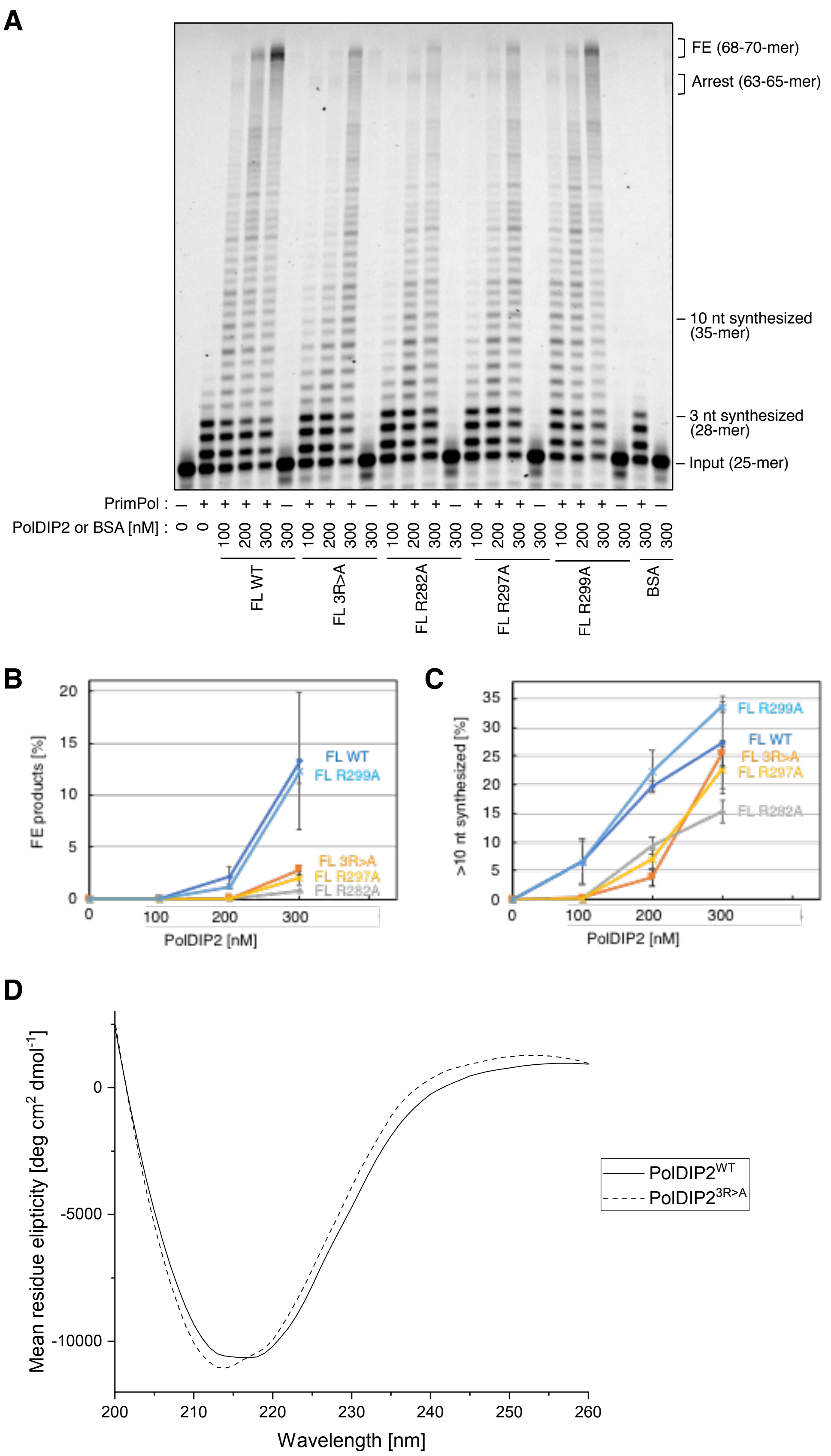

Supplementary Figure S6. The PolDIP2 Arg-cluster is required for PrimPol stimulation. (A) Primer extension experiments using 15 nM of 5'-TET-labeled

primer/template DNA and 150 nM of WT PrimPol in the presence of the indicated amounts of PolDIP2 variants. Quantification of (B) full extension and (C) >10 nt synthesized DNA products. These experiments were performed twice, and averages with standard deviations are shown with error bars. (D) Circular dichroism spectra of FL-PolDIP2 WT and the arginine cluster triple mutant 3R>A.
